# Evidence for a Functional *O*-Linked *N*-Acetylglucosamine (*O*-GlcNAc) System in the Thermophilic Bacterium Thermobaculum terrenum*

**DOI:** 10.1074/jbc.M115.689596

**Published:** 2015-10-21

**Authors:** Adam Ostrowski, Mehmet Gundogdu, Andrew T. Ferenbach, Andrey A. Lebedev, Daan M. F. van Aalten

**Affiliations:** From the ‡ Division of Molecular Microbiology and; § Medical Research Council Protein Phosphorylation and Ubiquitylation Unit, School of Life Sciences, University of Dundee, Dow Street, DD1 5EH Dundee, Scotland, United Kingdom and; ¶ Science Technology Facilities Council, Rutherford Appleton Laboratory, Didcot OX11 0FA, United Kingdom

**Keywords:** electron microscopy (EM), enzyme kinetics, glycosyltransferase, Gram-positive bacteria, O-GlcNAcylation, O-linked N-acetylglucosamine (O-GlcNAc), O-linked N-acetylglucosamine (O-GlcNAc) transferase (OGT), protein structure, Thermobaculum terrenum

## Abstract

Post-translational modification of proteins is a ubiquitous mechanism of signal transduction in all kingdoms of life. One such modification is addition of *O*-linked *N*-acetylglucosamine to serine or threonine residues, known as *O*-GlcNAcylation. This unusual type of glycosylation is thought to be restricted to nucleocytoplasmic proteins of eukaryotes and is mediated by a pair of *O*-GlcNAc-transferase and *O*-GlcNAc hydrolase enzymes operating on a large number of substrate proteins. Protein *O*-GlcNAcylation is responsive to glucose and flux through the hexosamine biosynthetic pathway. Thus, a close relationship is thought to exist between the level of *O*-GlcNAc proteins within and the general metabolic state of the cell. Although isolated apparent orthologues of these enzymes are present in bacterial genomes, their biological functions remain largely unexplored. It is possible that understanding the function of these proteins will allow development of reductionist models to uncover the principles of *O*-GlcNAc signaling. Here, we identify orthologues of both *O*-GlcNAc cycling enzymes in the genome of the thermophilic eubacterium *Thermobaculum terrenum.* The *O*-GlcNAcase and *O*-GlcNAc-transferase are co-expressed and, like their mammalian orthologues, localize to the cytoplasm. The *O*-GlcNAcase orthologue possesses activity against *O*-GlcNAc proteins and model substrates. We describe crystal structures of both enzymes, including an *O*-GlcNAcase·peptide complex, showing conservation of active sites with the human orthologues. Although *in vitro* activity of the O-GlcNAc-transferase could not be detected, treatment of *T. terrenum* with an *O*-GlcNAc-transferase inhibitor led to inhibition of growth. *T. terrenum* may be the first example of a bacterium possessing a functional *O*-GlcNAc system.

## Teaser

**Background:** Protein *O*-GlcNAcylation is essential for function and stability of many proteins in metazoa and is essential for development.

**Results:** *Thermobaculum terrenum* encodes a functional *O*-GlcNAc hydrolase and a conserved *O*-GlcNAc-transferase.

**Conclusion:** *T. terrenum* is the first known bacterium to possess the components for a functional *O*-GlcNAc system.

**Significance:** *T. terrenum* could become a reductionist model to study protein *O*-GlcNAcylation on an organism level.

## Introduction

Post-translational modifications of proteins are essential for cell signaling and regulation of cell biological processes. Probably the best understood type of such modification is protein phosphorylation, which can affect the conformation of the modified protein and as a result its activity, localization, or association with other proteins (for reviews, see Refs. 1 and 2). Conserved in all domains of life, protein phosphorylation is governed by a plethora of kinases and reciprocal phosphatases (3). These enzyme pairs are characterized by a high specificity for a small number of target proteins that they modify. In contrast, modification of cytoplasmic proteins with a single *O*-linked *N*-acetylglucosamine (*O*-GlcNAc)^4^ is a regulatory post-translational modification that is dependent on a single *O*-GlcNAc-transferase (OGT), which transfers the *O*-GlcNAc moiety onto target proteins, and *O*-GlcNAcase (OGA), which can reverse this process (4). This unusual type of protein glycosylation occurs exclusively on nucleocytoplasmic proteins in metazoa (5, 6). Since its discovery 30 years ago during a study of nucleoporins (7), over a thousand proteins have now been shown to be modified by *O*-GlcNAc (8). *O*-GlcNAc is a small, uncharged moiety, and the molecular basis of its impact on protein function is not well understood. However, there is evidence that protein *O*-GlcNAcylation can affect protein localization (9), activity (10), and stability (11). Additionally, the genetic disruption of *ogt* is lethal in vertebrates (12–18) and *Drosophila* (19). Given that protein *O*-GlcNAcylation occurs on serine and threonine residues, it has been suggested to show a degree of interplay with phosphorylation and that *O*-GlcNAcylation controls a number of signal transduction pathways (20).

**FIGURE 1.**
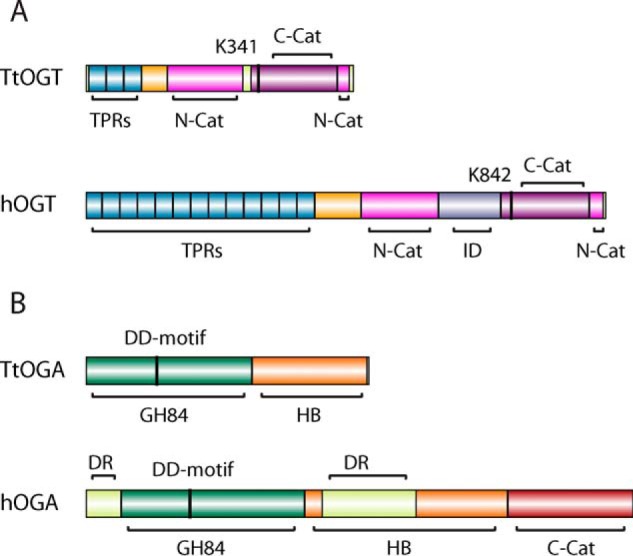
**Domain organization of human OGT (*A*) and OGA (*B*) enzymes and their orthologues from *T. terrenum*.** *A*, the TPR repeats of OGT are illustrated in *dark blue*, and the N- (*N-Cat*) and C-terminal (*C-Cat*) catalytic lobes of the GT41 family domain are shown in *pink* and *purple*, respectively. The catalytic lysine residues are marked with *lines*. The disordered regions are in *pale green*, and the intervening domain of hOGT is in *lilac. ID*, intervening domain. *B*, the catalytic *O*-GlcNAcase (GH84) domain is shown in *dark green*, the helical bundle domain is shown in *orange*, and the histone acetyltransferase domain of hOGA is shown in *dark red. HB*, helical bundle domain; *DR*, disordered region.

A typical eukaryotic OGT enzyme comprises an N-terminal tetratricopeptide repeat domain, which is required for interaction with some of the substrate proteins, and a C-terminal catalytic domain formed by two lobes separated by an intervening domain of unknown function (Fig. 1*A*) (21–23). OGT uses a form of substrate-assisted catalysis to transfer *N*-acetylglucosamine from the sugar-nucleotide donor UDP-GlcNAc onto specific serine or threonine residues of the substrate (24). *O*-GlcNAcase is a glycoside hydrolase responsible for hydrolysis of the link between the modified protein and the *O*-GlcNAc moiety (25). In metazoa, OGA consists of a glycoside hydrolase catalytic domain and a putative acetyltransferase domain(Fig. 1*B*) (26–31), although the relationship between the *O*-GlcNAcase and acetyltransferase activities of this enzyme is not understood.

One of the key questions in the *O*-GlcNAc signaling field is how two single enzymes, OGA and OGT, can together build a dynamic and inducible *O*-GlcNAc proteome of over a thousand *O*-GlcNAc proteins, whereas over 600 kinases/phosphatases are needed to carefully regulate site-specific protein phosphorylation in response to extracellular cues. Elucidating this essential mechanism is challenging in the model organisms where the greatest progress in understanding this modification has been achieved to date (*i.e.* mouse and *Drosophila*) and where OGT knock-outs are unfortunately lethal (19, 32). Thus, discovery of a much simpler, reductionist model system to study the basic mechanisms of *O*-GlcNAc signaling would be of considerable benefit.

*O*-GlcNAcylation is predominantly thought of as restricted to metazoa as OGT and OGA were initially identified across the Animalia kingdom (20). Two OGT orthologues, SPINDLY and SECRET AGENT, were subsequently identified in plants and are implicated in the gibberellin signaling pathway (33, 34). These two OGTs show a level of functional redundancy, and disruption of both genes is lethal (34). Furthermore, SECRET AGENT was shown to self-*O*-GlcNAcylate *in vitro* when expressed in *Escherichia coli* (34). However, other modified plant proteins remain to be identified, and there is currently no evidence of a functional OGA homologue in plant genomes.

Strikingly, many prokaryotic genomes of various genera appear to encode orthologues of both OGT and OGA. Some of these orthologues have been widely used in structural and enzymatic approaches to understand the molecular mechanism of *O*-GlcNAc transfer and hydrolysis (21, 26, 27, 35–38) despite the lack of any functional insight into their physiological roles. Several are secreted pathogenicity factors, like the NagJ from *Clostridium perfringens* (39) (hereafter *Cp*OGA), precluding a role in modulating intracellular *O*-GlcNAc signaling. A noteworthy exception is the recently identified OGT homologue found in the cyanobacterium *Synechococcus elongates* that appears to be involved in phosphorus retention within the cell, and genetic disruption causes the cells to aggregate (40). The underlying biological mechanisms of these phenotypes are not understood, and the organism lacks a predicted OGA homologue, precluding the existence of a dynamic *O*-GlcNAc proteome in this organism. It is possible that the single *S. elongates* OGT resembles the *O*-GlcNAcylation system found in plants.

In this report, we describe the identification of the first complete putative bacterial protein *O*-GlcNAcylation system, found in the soil thermophile *Thermobaculum terrenum* (41). By means of protein sequence searches, we identified orthologues of both OGT and OGA in this organism. We show that both proteins are expressed in *T. terrenum* under laboratory conditions and that both proteins are retained in the cytoplasm. The OGA orthologue is active *in vitro* on both a synthetic substrate and *O*-GlcNAc proteins, and treatment of *T. terrenum* with an OGT-specific inhibitor leads to growth inhibition. Unfortunately, throughout our experimental procedures, we were unable to identify proteins modified by the OGT homologue or detect *in vitro* activity of the recombinant protein. Finally, we use crystal structures of both enzymes to demonstrate conservation of the catalytic machinery, suggesting that this may represent a *bona fide O*-GlcNAc system orthologous to that found in metazoa.

## Experimental Procedures

### 

#### 

##### Bacterial Strains and Growth Conditions

*T. terrenum* strain YNP1 was obtained from ATCC. *T. terrenum* was routinely maintained at 65 °C with agitation in NYZ broth (10 g of casamino acids (Thermo Fisher), 5 g of yeast extract (Merck), 5 g of NaCl/liter) solidified with 0.8% Gelzan CM Gelrite (Sigma-Aldrich) when necessary. *T. terrenum* cells were streaked from a glycerol stock onto an NYZ plate and incubated at 65 °C for 5 days. A single colony was inoculated into 5 ml of NYZ broth supplemented with 0.2% glucose, and the starter culture was incubated at 65 °C for 2 days with vigorous agitation and used to inoculate experimental cultures. *Bacillus subtilis* strain 168 (Marburg) was routinely maintained and propagated in LB medium (10 g of Bacto tryptone (BD Biosciences), 5 g of yeast extract (Merck), 10 g of NaCl/liter). *E. coli* was routinely maintained in LB broth supplemented with 100 μg/ml ampicillin as required at 37 °C.

##### Molecular Cloning

Primers and plasmids used in this work are listed in Table 1. The coding frames of *Tter_2822* and *Tter_0116* genes were amplified using appropriate primer pairs from the genomic DNA of *T. terrenum* prepared using phenol/chloroform extraction. The amplified fragments were cloned into pGEX-6P-1 vector (GE Healthcare) using a restriction-free approach (42). Point mutations were introduced by site-directed mutagenesis using primers listed in Table 1 and verified by sequencing. All plasmids were cloned and maintained in *E. coli* DH5α.

**TABLE 1:**
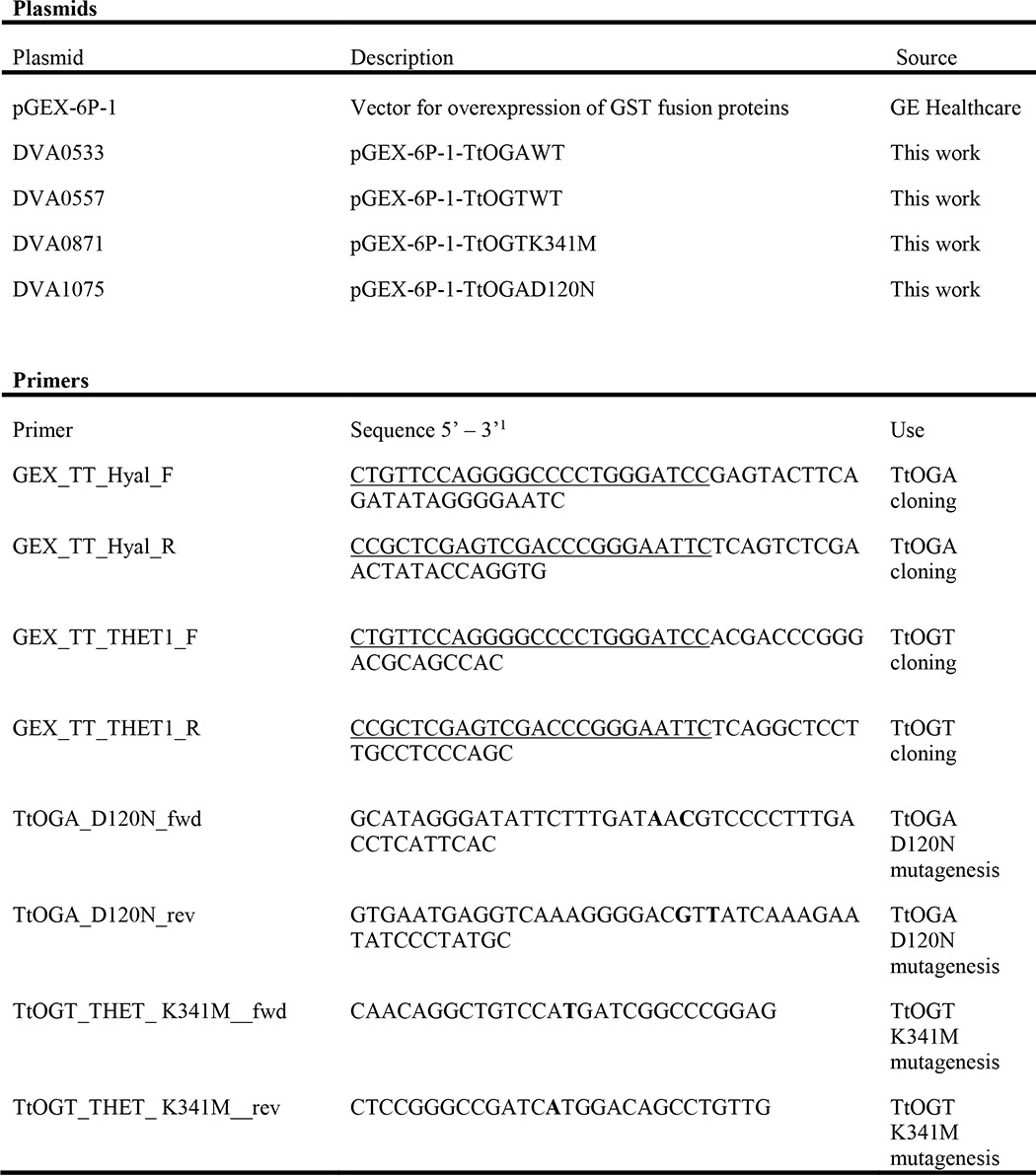
**Plasmids and primers**

^1^ Restriction-free cloning primer fragments homologous to the vector are underlined. The bases for site-directed mutagenesis substitutions are in bold.

##### Protein Purification and Antibody Production

Full-length recombinant *Tt*OGT and *Tt*OGA proteins were expressed as N-terminally GST-tagged fusions in *E. coli* BL21. Transformed strains were grown in autoinduction medium at 37 °C with agitation until *A*_600_ 0.3 at which point the temperature was lowered to 18 °C and incubation was continued overnight. Cells were harvested by centrifugation at 4 °C (35 min 4,500 × *g*). The cell pellets were resuspended in lysis buffer (50 mm HEPES, pH 7.5, 250 mm NaCl, 0.5 mm tris(2-carboxyethyl)phosphine) supplemented with 0.1 mg/ml DNase I and protease inhibitor mixture (1 mm benzamidine, 0.2 mm PMSF, 5 mm leupeptin) and disrupted using a continuous flow cell disruptor (three passes, 15,000 p.s.i.). After removing the cell debris (45 min, 30,000 × *g*), the supernatant was subjected to glutathione affinity chromatography using GSH-Sepharose beads (GE Healthcare) according to the manufacturer's instructions, and the desired product protein was liberated using PreScission protease (GE Healthcare). The cleaved protein was concentrated using centrifugal concentrators (Sartorius) and loaded onto a 300-ml prepacked Superdex^TM^ 75 column (GE Healthcare) equilibrated with lysis buffer. The protein peak was pooled and concentrated to 10 mg/ml for *Tt*OGT and 60 mg/ml for *Tt*OGA and used fresh in further experiments.

For the purpose of raising polyclonal antibodies, samples of the purified proteins were submitted to Dundee Cell Products for antibody production in rabbits. The antibodies were affinity-purified against the full-length purified proteins as described previously (43).

##### Analysis of Growth and Protein Localization in T. terrenum

To establish *T. terrenum* growth kinetics, 50-ml cultures were inoculated to an *A*_600_ of 0.025 from the starter cultures. The *A*_600_ of cultures was measured twice a day. To identify localization of *Tt*OGT and *Tt*OGA, 10-ml samples were removed from each culture, and the cell pellet was separated from the medium supernatant by centrifugation for 10 min at 4,000 × *g*. The decanted supernatant was filtered through a 0.2-μm syringe filter to remove any unpelleted cells. The cells were lysed by sonication in 500 μl of PBS, and the medium fraction was concentrated using VivaSpin 20 10-kDa-molecular mass cutoff spin concentrators (Sartorius) to 200 μl. To remove medium contaminants, the concentrated fraction was precipitated with methanol/chloroform and dissolved in 100 μl of PBS with 1% SDS, yielding a 400-fold concentration factor. The concentration of total protein in each of the samples was estimated by Coomassie staining of the SDS-PAGE gel. Standardized samples were resolved by10% SDS-PAGE. The gel was blotted onto nitrocellulose membrane using a Novex Semi-Dry Blotter, and the membranes were blocked in 3% milk in TBS, 0.1% Tween 20. The primary purified antibodies against *Tt*OGT (dilution, 1:100), *Tt*OGA (dilution, 1:1,000), and *B. subtilis* RpoD (44) (dilution, 1:1000) were incubated with the membranes overnight at 4 °C and detected with HRP-conjugated anti-rabbit secondary antibodies.

To assess the effects of peracetylated 5S-GlcNAc (Ac_4_-5S-GlcNAc) on growth of *T. terrenum*, the cells were inoculated to *A*_600_ 0.025 in 5 ml of NYZ broth supplemented with 0.2% glucose and 15–1,000 μm Ac_4_-5S-GlcNAc prepared in 100 μl of DMSO. Controls of cells treated with 100 μl of pure DMSO and untreated cells were included. The growth of cells was monitored by removing 100-μl samples and *A*_600_ measurement over the course of 92 h with sampling every 24 h from the moment of inoculation.

##### Enzymology

Steady-state kinetics of wild type and mutant *Tt*OGA were determined using the fluorogenic substrate 4-methylumbelliferyl-*N*-acetyl-β-d-glucosaminide (4MU-GlcNAc; Sigma). 50-μl reaction mixtures contained 0.2 nm enzyme in TBS buffer supplemented with 0.1 mg/ml BSA and 56–1,600 μm substrate in 2% DMSO. The fluorescence of the product, 4-methylumbelliferone (4MU), was quantified using an FLX 800 microplate fluorescence reader (Bio-Tek) with excitation and emission wavelengths of 360 and 460 nm, respectively. Experiments were performed in triplicate. Results were corrected for the background emission from the BSA, buffer, and the 4MU-GlcNAc, and the background-corrected data were fitted to the Michaelis-Menten equation using GraphPad Prism 5.0.

To determine the IC_50_ of GlcNAcstatin G, 4 pm wild type *Tt*OGA enzyme was incubated with the inhibitor (0.04–2,470 nm) for 1 min prior to starting the reaction by addition of the substrate. The substrate concentration was constant and equivalent to the *K_m_* determined from steady-state kinetics (90 μm). IC_50_ values were obtained by fitting the background-corrected fluorescence intensity data to a four-parameter equation for dose-dependent inhibition using GraphPad Prism 5.0. *K_i_* values were obtained from the conversion of the IC_50_ values using the Cheng-Prusoff equation: *K_i_* = IC_50_/(1 + [S]/*K_m_*).

##### In Vitro Deglycosylation of O-GlcNAc-Human TAK1-binding Protein 1 (hTab1) and Detection of O-GlcNAc

To incorporate the *O*-GlcNAc moiety onto recombinant hTab1, reactionmixtures containing 4.6 μm hTab1^7–402^ protein, 1.25 μm hOGT^312–103^, and 3.7 mm UDP-GlcNAc in a reaction buffer (50 mm Tris, pH 7.5, 1 mm DTT) were incubated for 1 h at room temperature. The glycosylated hTab1 was supplemented with *Cp*OGA^31–618^ (1 μm), increasing concentrations of wild type *Tt*OGA (1, 5, and 15 μm), D120N mutant of *Tt*OGA (15 μm), or wild type *Tt*OGA preincubated with GlcNAcstatin G (100 μm) and incubated for 6 h at 37 °C. The proteins were resolved by SDS-PAGE (12% gels) and transferred onto nitrocellulose membranes. The membranes were probed with an *O*-GlcNAc-specific antibody (RL-2, Abcam) followed by an IR800-labeled secondary antibody and analyzed using a LI-COR Odyssey scanner and associated quantification software.

##### Protein Crystallography

To crystallize *Tt*OGT, sitting drops containing 200 nl of reservoir solution (12.5% poly(acrylic acid sodium salt) 2100, 0.5 m (NH_4_)_3_PO_4_), 0.1 m SrCl_2_, and 200 nl of 5 mg/ml *Tt*OGT^K341M^ and 0.9 mm UDP-5S-GlcNAc in 50 mm HEPES, pH 7.5, 250 mm NaCl, 0.5 mm tris(2-carboxyethyl)phosphine equilibrated against 65 μl of reservoir solution gave small, tetragonal crystals in 3–4 days at 22 °C. These crystals were converted into seed stocks and used to nucleate crystal growth at the same conditions but with 10 mm UDP instead of 0.9 mm UDP-5S-GlcNAc. These crystals were cryoprotected by 2-s immersion in a 20% glycerol solution before flash freezing in liquid nitrogen.

To crystallize *Tt*OGA, sitting drops containing 200 nl of reservoir solution (38% PEG 4000, 400 mm sodium acetate, 0.1 m Tris-HCl, pH 8.5) and 200 nl of 40 mg/ml *Tt*OGA^D120N^ and 7.2 mm hTab1-*O*-GlcNAc peptide (^392^VPY**gS**SAQ^398^ where **gS** is GlcNAcylated serine) in 50 mm HEPES, pH 7.5, 250 mm NaCl, 0.5 mm tris(2-carboxyethyl)phosphine equilibrated against 65 μl of reservoir solution gave small bipyramidal crystals in 3–4 days at 22 °C. Crystals were flash- frozen in liquid nitrogen without prior cryoprotection.

The diffraction data of *Tt*OGT and *Tt*OGA crystals were collected at the Diamond Synchrotron beamline ID04 and the European Synchrotron Radiation Facility beamline ID23-1, respectively. *Tt*OGA and *Tt*OGT data were processed with XDS (45) and scaled to 2.06 and 2.80 Å, respectively, using SCALA (46). The *Tt*OGA structure was solved using molecular replacement (Protein Data Bank code 2XSA (28)), and automated model building was performed using ARP/wARP (47). The resulting model was then manually completed and refined with Coot (48) and REFMAC (49). The structure of *Tt*OGT was solved by molecular replacement (Protein Data Bank code 3PE3 (22)), and initial model building was performed with Buccaneer (50) aided by 4-fold NCS averaging. The space group ambiguity induced by pseudotranslation was resolved using Zanuda (51). The resulting model was then manually completed and refined with Coot (48) and REFMAC (49).

##### Electron Microscopy

For transmission electron microscopy, *T. terrenum* was grown in 25 ml of NYZ supplemented with 0.2% glucose and 100 μl of DMSO (vehicle control) or 500 μm Ac_4_-5S-GlcNAc in 100 μl of DMSO for 62 h. A 10-ml sample was removed and fixed by addition of glutaraldehyde to a final concentration of 2.5% and incubation for 1 h on ice. The cells were pelleted and processed as described previously (41). Transmission electron microscopy was performed using a JEOL JEM-1200EX electron microscope, and the images were captured on electron-sensitive film.

##### Data Analysis and Image Processing

All enzyme activity and bacterial growth analysis was performed in Prism (GraphPad). Enzyme domain organization figures were prepared in DOG (GPS) (52), and sequence alignments were prepared using Clustal Omega (53) and processed in ALINE (54). Protein structures were analyzed using PyMOL (The PyMOL Molecular Graphics System, Version 1.2r3pre, Schrödinger, LLC) and Coot (48). All figures were assembled in Adobe Illustrator CS5.1.

## Results

### 

#### 

##### T. terrenum Possesses Apparent Orthologues of Both OGT and OGA

**FIGURE 2.**
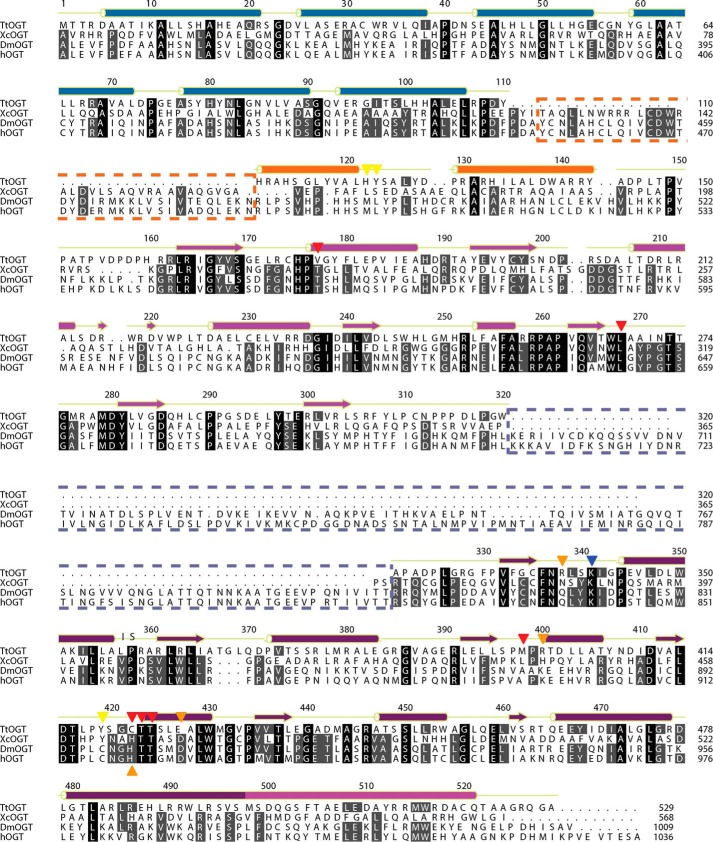
**Sequence alignment of *O*-GlcNAc-transferase homologues from *T. terrenum*, *X. campestris* (*Xc*), *D. melanogaster* (*Dm*), and *H. sapiens* (*h*).** Sequence numbering and secondary structure annotation are in accordance to the *T. terrenum* OGT structure. α-Helices are indicated by *barrels*, and β-sheets are indicated by *arrows* (TPRs, blue; TLR, *orange*; GT41-N, *pink*, and GT41-C, *magenta*). Insertions at the TLR and the intervening domain of hOGT are indicated with *dashed boxes* colored *orange* and *lilac*, respectively. The *blue triangle* marks the catalytic lysine, *red triangles* indicate the residues that form sequence-independent hydrogen-bonding interactions, *orange triangles* indicate residues that form side chain-specific interactions with UDP-GlcNAc, and *yellow triangles* indicate the residues that form the hydrophobic pocket in which the *N*-acetyl moiety of GlcNAc fits in hOGT.

**FIGURE 3.**
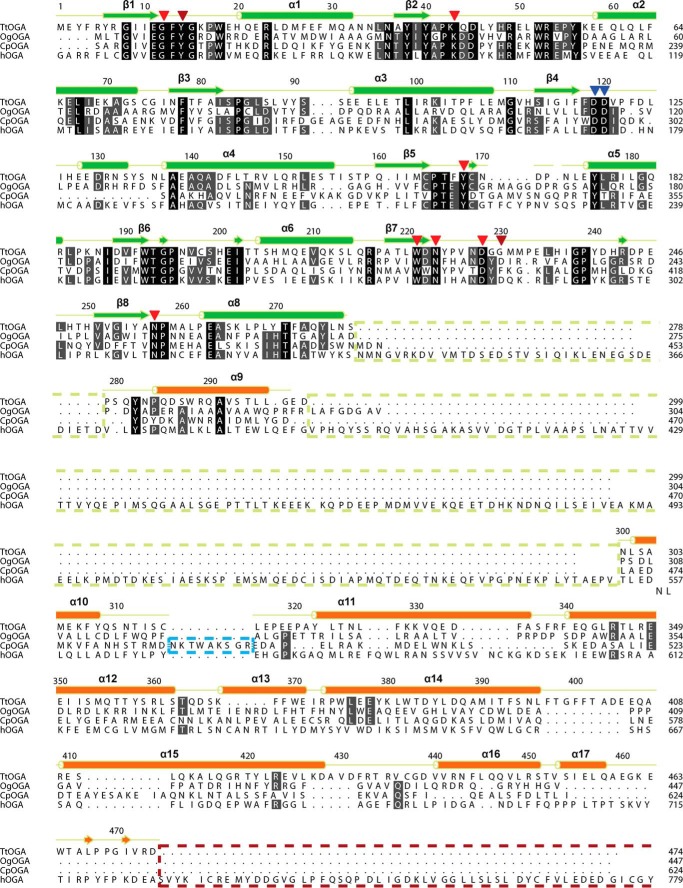
**Sequence alignment of OGA homologues from *T. terrenum*, *O. granulosus*, *C. perfringens*, and *H. sapiens* (*h*).** Sequence numbering and secondary structure annotation are in accordance to the *T. terrenum* OGA structure. α-Helices are indicated by *barrels*, and β-sheets are indicated by *arrows* (TIM barrel, *green*; helical bundle domain, *orange*). The disordered region and the histone acetyltransferase domain of hOGA are indicated with *dashed boxes* colored *pale yellow* and *dark red*, respectively. The extended α10-α11 loop of *Cp*OGA is marked with a *cyan dashed box*. The *blue triangle* marks the catalytic double aspartate motif, the *red triangles* indicate the residues that tether the *O*-GlcNAc moiety of the substrate, and the *dark red triangles* indicate the residues that play a role in recognition/positioning of the peptide in *Tt*OGA.

To identify candidate microorganisms harboring putative *O*-GlcNAc cycling enzymes, we searched the carbohydrate-active enzymes database CAZy (55) for species possessing members of both the glycosyltransferase 41 (GT41; OGT) and glycoside hydrolase 84 (GH84; OGA) families. The shortlist was then analyzed to exclude those species where either OGA or OGT possessed putative secretion signal peptides predicted with SignalP (56), leaving species putatively possessing complete intracellular *O*-GlcNAc cycling machinery. This analysis resulted in identification of a single species, *T. terrenum* YNP1, a Gram-positive thermophilic eubacterium isolated from soil near a hot spring in Yellowstone National Park (41). We identified the products of genes *Tter_2822* (hereafter *Ttogt*) and *Tter_0116* (hereafter *Ttoga*) as putative OGT and OGA orthologues, respectively. To validate these predictions, we aligned the translated sequences of the GT41 domains of *Tt*OGT against the known/putative *O*-GlcNAc-transferases from *Xanthomonas campestris* pv. *campestris* (35), *Drosophila melanogaster* (57), and *Homo sapiens* (58) (Figs. 1*A* and 2). The predicted tetratricopeptide repeat (TPR) region of *Tt*OGT aligns with the eukaryotic orthologues (Fig. 1*A*), and the presence of three TPRs at the N terminus of *Tt*OGT was separately confirmed using TPRpred software. Both compared bacterial OGT orthologues appear to lack the intervening domain of unknown function separating the two catalytic lobes of the eukaryotic OGTs. The sequences of the N- and C-terminal catalytic lobes of *Tt*OGT were 25 and 29% identical to that of hOGT, respectively, and the key catalytic residue Lys^341^ (Lys^842^ in hOGT (24)) is conserved (Fig. 2). Similarly, the sequence of *Tt*OGA was aligned against the protein sequences of (putative) OGA homologues from *Oceanicola granulosus* (28), *C. perfringens* (27), and *H. sapiens* (25) (Figs. 1*B* and 3). *Tt*OGA comprises the GH84 and helical bundle domains but lacks the putative histone acetyltransferase domain found in eukaryotic homologues (Fig. 1*A*). The sequence identity of the *Tt*OGA GH84 domain to that of human OGA (hOGA) was 36%, and the catalytic DD motif (Asp^119^, Asp^120^ in *Tt*OGA and Asp^174^, Asp^175^ in hOGA) is conserved (Fig. 3). We concluded from this analysis that the catalytic domain architecture between *T. terrenum* OGT/OGA and their human orthologues appears to be conserved.

##### TtOGT and TtOGA Are Intracellular Proteins

**FIGURE 4.**
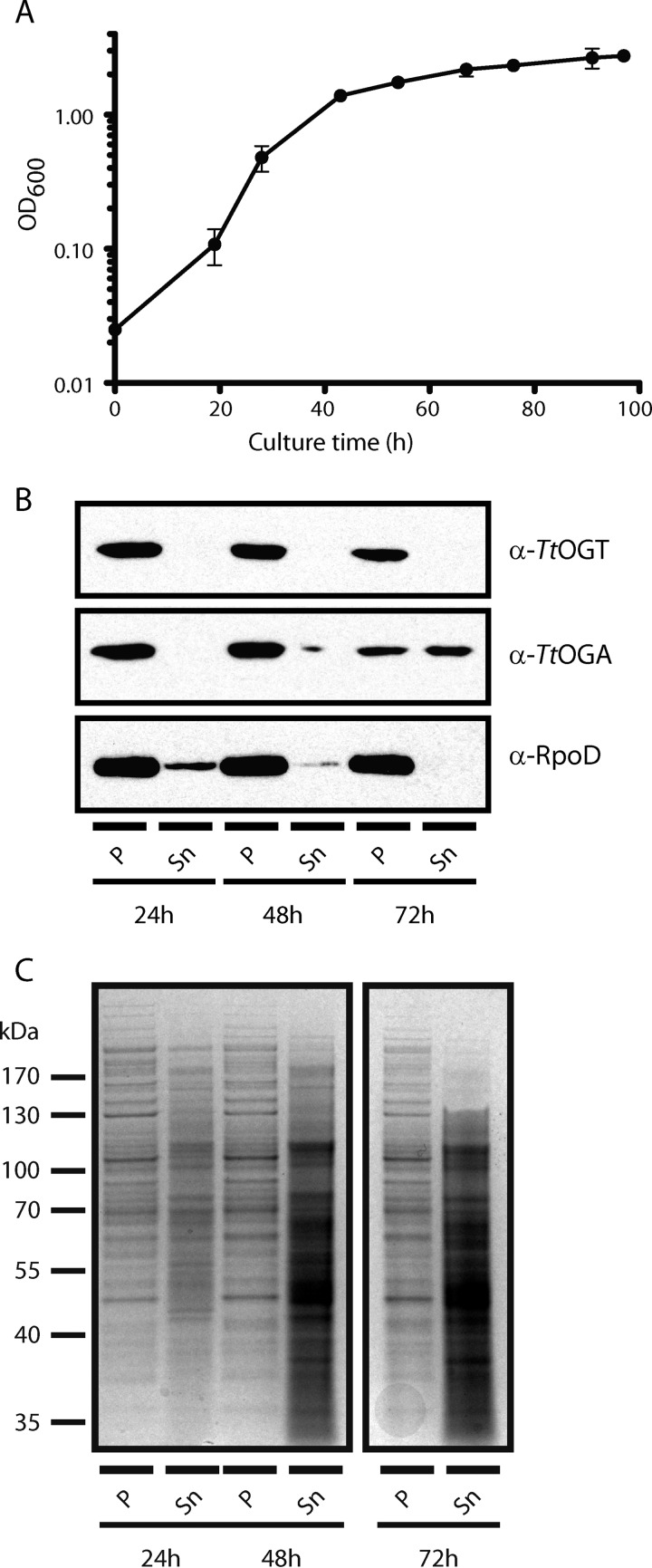
***Tt*OGT and *Tt*OGA are expressed throughout the exponential phase of growth and are intercellular proteins.** *A*, the growth of *T. terrenum* was followed in NYZ medium supplemented with 0.2% glucose for 92 h. The optical density of the cultures was measured at 600 nm. The curve shown is an average of three independent biological replicates. The *error bars* represent S.D. from the mean. *B*, the samples of the cell pellet (*P*) and filtered medium supernatant (*Sn*) taken at 24, 48, and 72 h postinoculation were analyzed for the presence of *Tt*OGT, *Tt*OGA, and housekeeping σ factor RpoD. The supernatant fraction was concentrated 400-fold prior to the analysis. *C*, loading control of the anti-*Tt*OGA and anti-*Tt*OGA Western blot. Identical samples of the cell pellet lysate and precipitated medium supernatant fractions were resolved by 10% SDS-PAGE and stained with InstantBlue. Images of all lanes were acquired from different parts of one gel.

The metazoan *O*-GlcNAc cycling enzymes are localized to the cytoplasmic and nuclear compartments of the cell. We investigated whether the *T. terrenum* orthologues were retained within the cell as suggested by the predicted lack of signal peptides and the absence of type IV and type VII secretion systems in the *T. terrenum* genome. To this end, we raised polyclonal antibodies against recombinant *Tt*OGT and *Tt*OGA expressed in *E. coli*. We optimized growth conditions of *T. terrenum* in NYZ broth supplemented with 0.2% glucose. In these conditions, the doubling time of the organism was close to 4.5 h in the exponential phase of growth with stationary phase reached within 90 h (Fig. 4*A*). We analyzed the cell pellet and the medium supernatant samples collected from early, mid-, and late exponential growth phases for the presence of *Tt*OGT and *Tt*OGA using the polyclonal antibodies. The samples were also probed for the σ^A^ (RpoD) transcription factor to control for (auto)lysis and release of intracellular components into the medium (Fig. 4*B*). The data show that *Tt*OGT and *Tt*OGA are predominantly found in the whole cell lysate. In the late exponential growth phase sample set (72 h), we observed some spillage of *Tt*OGA into the medium, although it should be noted that supernatants were concentrated 400-fold (see “Experimental Procedures” and Fig. 4*C*). We also noticed lower intensity bands corresponding to RpoD from the medium supernatant fractions taken at early and mid-exponential points, suggesting a degree of cell lysis during all stages of bacterial growth. From this analysis, we concluded that both *Tt*OGT and *Tt*OGA are synthesized throughout the exponential growth phase of *T. terrenum* and that these proteins are not actively secreted from the cell. Thus, these proteins are appropriately co-expressed and co-localized to form a putative *O*-GlcNAc cycling system.

##### TtOGA Is an Active O-GlcNAc Hydrolase

**FIGURE 5.**
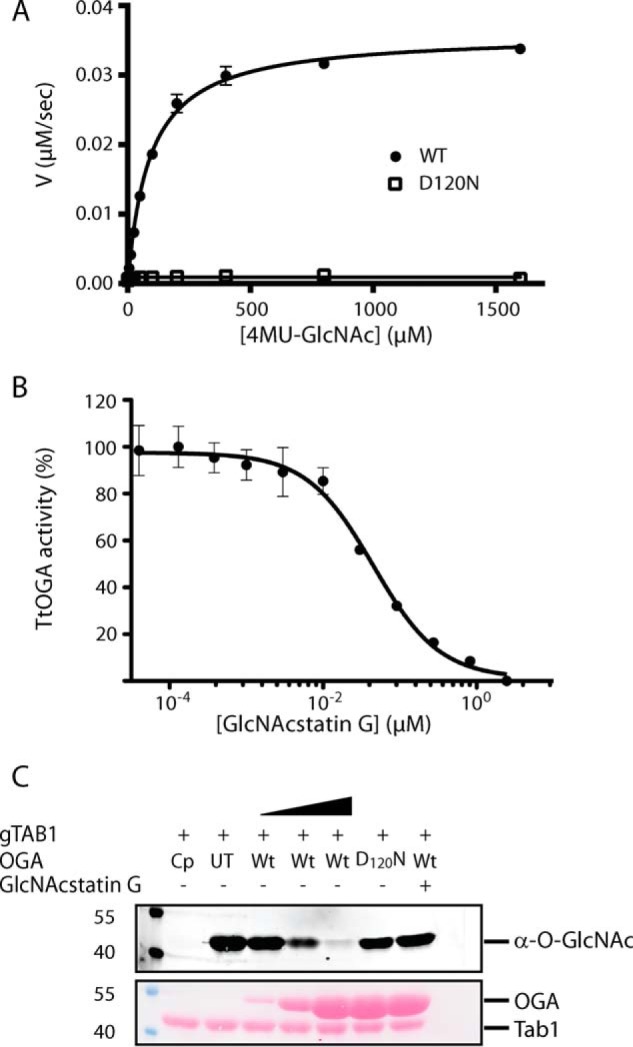
***Tt*OGA is an active *O*-GlcNAcase.** *A*, steady-state kinetics of the enzymatic activity of the wild type (*closed black circles*) and D120N (*open squares*) *Tt*OGA against the pseudosubstrate 4MU-GlcNAc. The initial velocities of the enzymatic reactions (in μm of product/s) in relation to the substrate concentration (in μm) are shown. *B*, wild type *Tt*OGA subjected to the inhibitor GlcNAcstatin G. Inhibition is shown as percentage of activity of the uninhibited enzyme as a function of inhibitor concentration in μm. The results are representative of three biological repeats, and the *error bars* represent S.D. *C*, *Tt*OGA *O*-GlcNAcase activity on glycosylated hTab1. *O*-GlcNAcylated hTab1 was treated with an increasing concentration (1, 5, and 15 μm) of the wild type *Tt*OGA (*WT*), the inactive D120N mutant (15 μm), the wild type enzyme (15 μm) preincubated with GlcNAcstatin G, or wild type *Cp*OGA (1 μm) (*Cp*) as a positive control. An untreated negative control is shown (*UT*). *O*-GlcNAcylated proteins were detected with *O*-GlcNAc-specific RL-2 antibody. Even loading was tested by staining of the membrane with Ponceau S. Bands corresponding to *Tt*OGA and hTab1 are indicated.

CAZy (55) classifies all *O*-GlcNAcases as members of GH84, which also includes a number of bacterial proteins, including *Tt*OGA. To verify whether *Tt*OGA is a *bona fide O*-GlcNAc hydrolase, the wild type enzyme (*Tt*OGA^WT^) together with two mutants (*Tt*OGA^D120N^ and *Tt*OGA^D228A^) were cloned and purified as GST fusions from *E. coli*. Using the structure and mutagenesis data of hOGA (28) and *Cp*OGA (27) as a guide, these mutations were designed to target the catalytic DD motif (the D120N mutation) and the substrate binding site (the D228A mutation). We tested the activity of these enzymes against the fluorogenic pseudosubstrate 4MU-GlcNAc. The *K_m_* of *Tt*OGA^WT^ against 4MU-GlcNAc was 90 μm with a *k*_cat_ of 180 s^−1^ (Fig. 5*A*). As expected, the activity of the *Tt*OGA^D120N^ mutant protein was significantly reduced (*k*_cat_ of 5 s^−1^)) in comparison with the wild type enzyme, whereas *Tt*OGA^D228A^ showed no detectable activity (Fig. 5*A*). To further investigate the *O*-GlcNAcase activity of *Tt*OGA, we utilized a well characterized small molecule OGA inhibitor, GlcNAcstatin G (38). This inhibitor showed a dose-dependent inhibition of the reaction catalyzed by *Tt*OGA^WT^ with a calculated *K_i_* of 18 nm (Fig. 5*B*).

Having shown that *Tt*OGA exhibits *in vitro* enzymatic parameters comparable with those of previously characterized OGAs, we next investigated whether *Tt*OGA is capable of removing *O*-linked GlcNAc from protein substrates. To this end, we utilized a well characterized substrate of hOGT, hTab1 (59). Purified recombinant hTab1 was first *O*-GlcNAcylated *in vitro* using recombinant hOGT^312–1023^ and subsequently incubated with wild type and inactive mutants of *Tt*OGA. The highly active bacterial OGA orthologue *Cp*OGA (27) was used as a positive control. Incubation of *O*-GlcNAcylated hTab1 with increasing concentrations of *Tt*OGA^WT^ led to a decrease of *O*-GlcNAcylation as detected by the *O*-GlcNAc-specific antibody RL-2 (Fig. 5*C*). A 60-min incubation with 15 μm *Tt*OGA^WT^ was sufficient to remove *O*-GlcNAc from hTab1 nearly entirely. This effect was ablated by addition of 100 μm GlcNAcstatin G or if catalytically deficient *Tt*OGA^D120N^ was used instead (Fig. 5*C*).

##### An OGT Inhibitor Is Bacteriostatic to T. terrenum

**FIGURE 6.**
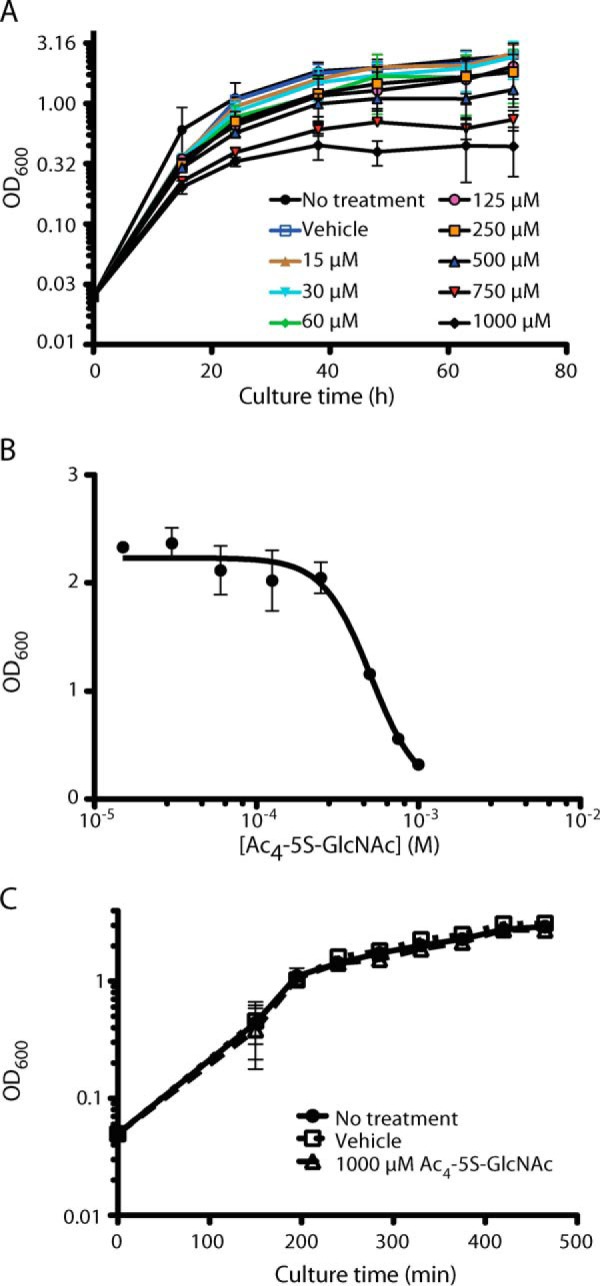
**Cell-permeable OGT inhibitor precursor Ac_4_-5S-GlcNAc is bacteriostatic to *T. terrenum*.** *A*, *T. terrenum* cells were treated with increasing concentrations of Ac_4_-5S-GlcNAc from 15 to 1,000 μm in 100 μl of DMSO, and the culture growth was followed for 72 h by measuring optical density at 600 nm. The resulting growth curves were compared with growth of the untreated culture as well as that treated with a vehicle control (100 μl of DMSO). *B*, the effect of Ac_4_-5S-GlcNAc on the growth of *T. terrenum* is dose-dependent. The half-maximal effective concentration of Ac_4_-5S-GlcNAc (509 μm) was calculated from the *A*_600_ values at the 62-h time point. The data are representative of three independent biological replicates. The *error bars* represent S.D. from the mean. *C*, Ac_4_-5S-GlcNAc treatment has no effect on growth of *B. subtilis.* The growth of *B. subtilis* without treatment (*black circles*), with treatment with vehicle control (100 μl of DMSO) (*open squares*), or with treatment with 1 mm Ac_4_-5S-GlcNAc in 100 μl of DMSO (*open triangles*) was measured every 45 min over a 500-min time course by measuring the optical density at 600 nm.

**FIGURE 7.**
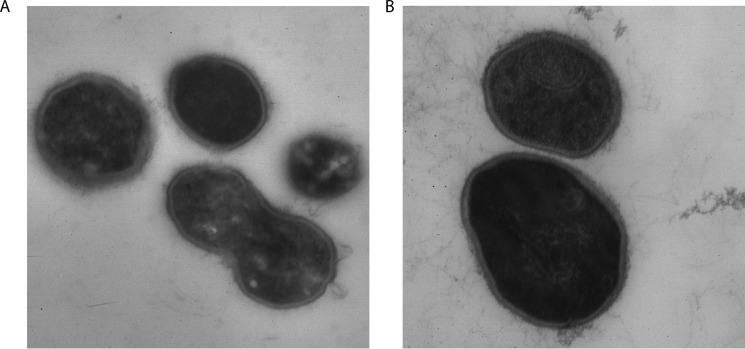
**Ac_4_-5S-GlcNAc does not affect the thickness of *T. terrenum* cell wall.** *T. terrenum* cells were grown for 62 h in the presence of DMSO (*A*) or 500 μm Ac_4_-5S-GlcNAc (*B*). The cells were fixed with glutaraldehyde and embedded in a paraffin block, and cross-sections were imaged by transmission EM at ×40,000 magnification.

We were interested in identifying *O*-GlcNAc-modified proteins in the proteome of *T. terrenum*. Initially, we used Western blotting with the *O*-GlcNAc-specific antibodies RL-2 and CTD110.6; however, this was unsuccessful. This was followed by electron transfer dissociation MS unbiased analysis of the proteome, which also did not identify reliable candidate proteins. We next attempted a genetic approach toward probing the function of *Tt*OGA/OGT. However, after numerous attempts at transforming *T. terrenum* by electroporation with replicating or integrating plasmids conveying antibiotic or heavy metal resistance, we were unable to genetically manipulate *T. terrenum*, thus precluding genetic strategies toward probing the functions of the OGA and OGT orthologues. As an alternative, we used a chemical approach. UDP-5S-GlcNAc is an analogue of the sugar-nucleotide donor UDP-GlcNAc that is poorly transferred onto acceptor substrates by hOGT (60). Due to the unique mechanism of action of OGT (21), this inhibitor is believed to be specific to OGT and to not inhibit other UDP-GlcNAc-dependent transferases (60). This inhibitor is made by the intracellular hexosamine biosynthetic pathway upon feeding cells with the cell-penetrant precursor Ac_4_-5S-GlcNAc (60). We supplemented cultures of *T. terrenum* with a range of concentrations of the precursor and followed the growth over a course of 92 h (Fig. 6*A*). Strikingly, we observed a decrease in the growth rate and maximal optical density of the culture in a dose-dependent manner. Using these data, we estimated the half-maximal effective concentration of Ac_4_-5S-GlcNAc to be 500 μm (Fig. 6*B*). However, it is possible that the growth defect observed in *T. terrenum* is due to an off-target effect of inhibition of a glycosyltransferase that is exclusive to the Bacteria kingdom. To explore this possibility, we took a 2-fold approach. First, we assessed whether the synthesis of peptidoglycan, the main *N*-acetylglucosamine sink in a bacterial cell, is affected by treatment with Ac_4_-5S-GlcNAc. We assessed the thickness of cell wall peptidoglycan by cross-sectioning transmission EM. No visible difference was observed between the morphology of the cells treated with Ac_4_-5S-GlcNAc and those treated with a vehicle control, suggesting that this compound does not target glycosyltransferases involved in peptidoglycan biosynthesis (Fig. 7). Second, we tested the effects of Ac_4_-5S-GlcNAc on growth of *B. subtilis*, another Gram-positive bacterium. In this case, even treatment with 1 mm Ac_4_-5S-GlcNAc, the bacteriostatic concentration for *T. terrenum*, had no significant effect on growth of *B. subtilis* (Fig. 6*C*). Thus, it seems likely that Ac_4_-5S-GlcNAc affects a mechanism specific to *T. terrenum* rather than a well conserved bacterial pathway with *Tt*OGT as a possible target.

In an attempt to verify that the effects of treatment with Ac_4_-5S-GlcNAc were indeed due to inhibition of *Tt*OGT, we explored *in vitro* activity of the recombinant protein. As putative target proteins, we used a well established hTab1-derived peptide (24) as well as a library of degenerate peptides. Unfortunately, we were unable to detect *in vitro* activity of *Tt*OGT against any of the substrate peptides.

##### TtOGT and TtOGA Crystal Structures Reveal Catalytically Competent Active Sites

**FIGURE 8.**
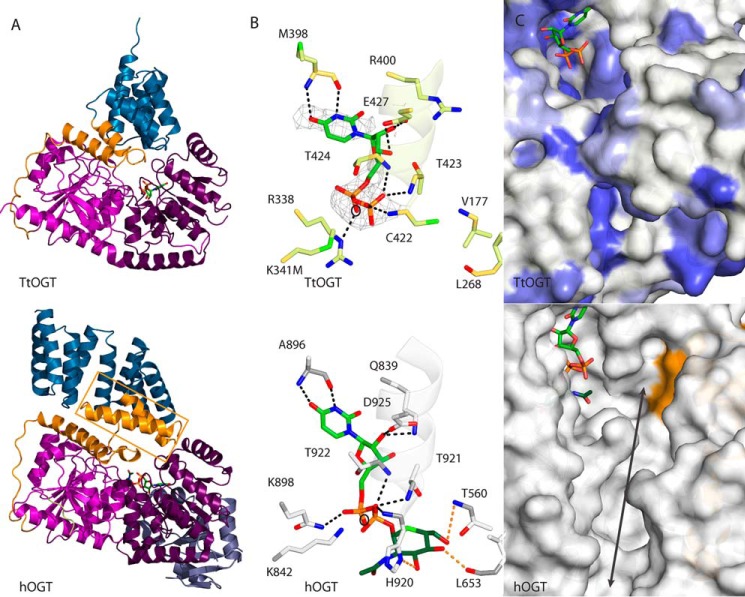
**Structural characterization of *Tt*OGT.** *A*, schematic representation of *Tt*OGT depicting the secondary structure elements. Presented in the *lower panel* is hOGT (Protein Data Bank code 4AY6). The region of the hOGT TLRs that is absent in *Tt*OGT is demarcated with an *orange box*. Coloring is as in Fig. 1. *B*, *Tt*OGT active site with the unbiased positive density for UDP. UDP is depicted in *stick* representation. Interacting residues of *Tt*OGT are labeled, and the interactions are depicted by *dashed lines*. The α-helical electrostatic dipole is also shown in schematic representation. Presented in the *lower panel* is a complex of hOGT with UDP-5S-GlcNAc (Protein Data Bank code 4AY6). The *orange dashed lines* represent the interactions between hOGT and the GlcNAc moiety. The catalytic base is marked by a *circle*. UDP, *light green*; GlcNAc, *dark green. C*, *Tt*OGT active site conservation. Surface residues that are identical in *Tt*OGT and hOGT are colored *dark blue*, and functionally conserved residues are colored *light blue*. Presented in the *lower panel* is the structure of hOGT (Protein Data Bank code 4AY6). The surface region corresponding to the hOGT TLRs that are absent in *Tt*OGT is colored *orange*. The protein substrate docking groove of hOGT is indicated by an *arrow*.

**FIGURE 9.**
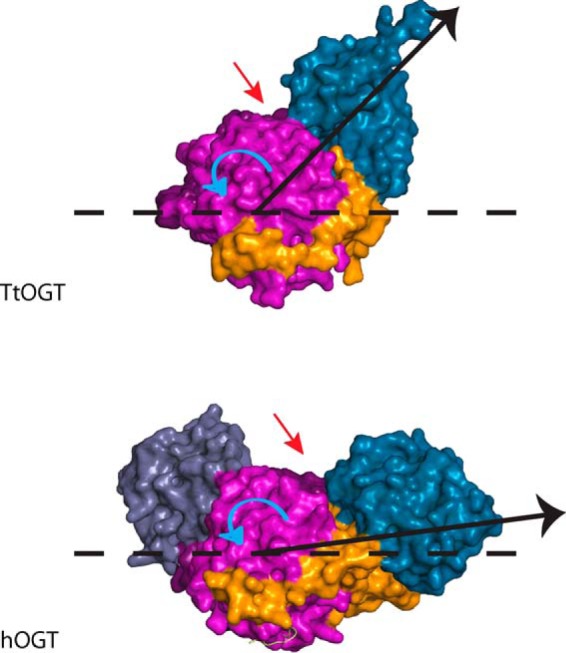
**The direction of the TPR superhelix.** Surface representations of *Tt*OGT (*top*) and hOGT (*bottom*) are shown. The TPR repeats of OGT are illustrated in *dark blue*, and the N- and C-terminal catalytic lobes of the GT41 family domain are shown in *pink* and *purple*, respectively. The *black dashed line* represents the plane of the GT41 domain from the cross-section, the *black arrow* represents the direction of the superhelix relative to the plane of GT41 domain, the *red arrow* indicates the active site groove, and the *curved blue arrow* highlights the angle between the direction of the TPR superhelix and the plane of the GT41 domain.

To investigate the catalytic machinery and conservation of substrate/product binding modes, we determined the crystal structures of *Tt*OGT and *Tt*OGA. *Tt*OGT was co-crystallized with the product of *O*-GlcNAc transfer, UDP. The structure, solved by molecular replacement and refined against synchrotron diffraction data of 2.8 Å (Table 2), revealed an ordered N-terminal TPR domain and a bilobal catalytic domain at the C terminus (Fig. 8*A*). The *Tt*OGT TPR domain itself displays good structural similarity with that of hOGT (r.m.s.d. = 1.3 Å for 106 C^α^ atoms). However, a deletion at the region previously designated as the tetratricopeptide-like region (TLR) (35) (Figs. 1*A* and 2) results in a major change in the orientation of the TPR domain relative to the GT41 catalytic domain compared with hOGT (Figs. 8*A* and 9). This more direct/rigid fusion of the *Tt*OGT TPRs to the GT41 domain compared with that of hOGT creates a relatively narrow groove for the protein substrates to bind near the active site (Figs. 8*C* and 9). It is possible that this could explain the lack of detected activity against protein substrates observed in all *in vitro* assays we explored. However, the GT41 domain of *Tt*OGT is similar to that of hOGT (r.m.s.d. = 1.5 Å for 303 C^α^ atoms) despite the absence of the intervening domain of unknown function that is positioned between the two lobes of this domain in hOGT (Figs. 1*A* and 8*A*). The human enzyme achieves catalysis by inducing a unique conformation of the donor substrate (24). Catalysis of *O*-GlcNAc transfer is facilitated by the pro-*R*_P_ oxygen of the α-phosphate acting as the catalytic base. It is proposed that the presence of an oxyanion hole formed by backbone amides of His^920^, Thr^921^, and Thr^922^ (equivalent to Cys^422^, Thr^423^, and Thr^424^ in *Tt*OGT; maximum atomic shift of 1.7 Å), an α-helical electrostatic dipole, and the evolutionarily conserved Lys^842^ (Lys^341^in *Tt*OGT) function together to ensure the correct positioning of the donor substrate and to stabilize the negative charge developing on the leaving group (24). Conservation of all these components in *Tt*OGT (Fig. 8*B*) implies that this may be a catalytically competent enzyme. Furthermore, superposition of a hOGT·UDP-5S-GlcNAc complex (Protein Data Bank code 4AY6) onto the *Tt*OGT structure reveals the conservation of the additional interactions that are required to tether UDP-GlcNAc to the active site. There are six sequence-independent interactions, *i.e.* mediated via backbone amide nitrogens and oxygens, that are structurally conserved (maximum atomic shift of 1.7 Å between the interacting nitrogen or oxygen atoms) and four sequence-dependent hydrogen-bonding interactions that are also conserved (maximum atomic shift of 1.7 Å between the interacting nitrogen or oxygen atoms) (Fig. 8*B*) despite a conservative substitution of His^920^ in hOGT with Cys^422^ in *Tt*OGT and substitution of Lys^898^ with Glu^427^ (Fig. 8*B*). Strikingly, the hydrophobic pocket formed by Met^501^, Leu^502^, and Cys^917^ at the hOGT active site, which fits the *N*-acetyl moiety of UDP-GlcNAc in the hOGT active site, is not apparent in the *Tt*OGT·UDP complex. It is possible that a conformational change is required to form this hydrophobic pocket and perhaps to reorient the TPRs to create an active site groove that is more permissive for docking of a protein substrate.

**TABLE 2:** **Merging, scaling, and refinement statistics** Values in parentheses represent outer shell reflections only.

	*Tt*OGA	*Tt*OGT
Space group	C 2	P 2_1_ 2_1_ 2_1_
Unit cell (Å)	*a* = 52.6, *b* = 132.5, *c* = 161.2	*a* = 70.19, *b* = 216.36, *c* = 216.40
Resolution (Å)	49.03–2.06 (2.13–2.06)	49.00–2.80 (8.85–2.80)
No. reflections	236,413 (22,742)	551,360 (80,160)
No. unique reflections	57,764 (5,569)	82,234 (11,826)
Redundancy	4.1 (4.1)	6.7 (6.8)
Mean (*I*/σ*I*)	12.8 (1.8)	12.6 (2.5)
Completeness (%)	99.6 (98.8)	100 (99.9)
*R*_merge_	0.071 (0.681)	0.13 (0.69)
*R*_work_/*R*_free_ (%)	20.1/23.6	21.8/24.6
r.m.s.d. bonds (Å)	0.07	0.09
r.m.s.d. angles (°)	1.2	1.4

**FIGURE 10.**
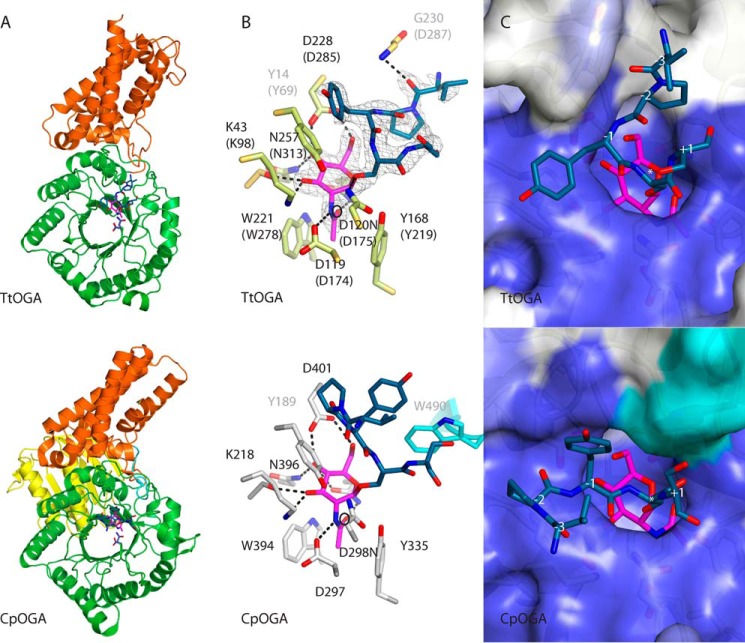
**Structural characterization of *Tt*OGA·hTab1-*O*-GlcNAc complex.** *A*, schematic representation of *Tt*OGA depicting the secondary structure elements. The DD motif is shown in *stick* representation. Presented in the *lower panel* is the *Cp*OGA crystal structure (Protein Data Bank code 2YDS). Coloring is as in Fig. 1. *B*, *Tt*OGA active site and the unbiased positive density for hTab1-*O*-GlcNAC peptide. hTab1-*O*-GlcNAC peptide is shown in *stick* representation. Interacting residues of *Tt*OGT are labeled (hOGA equivalents in *parentheses*), and the interactions are depicted by *dashed lines*. Presented in the *lower panel* is a complex of *Cp*OGA with the same ligand (Protein Data Bank code 2YDS). The α10-α11 loop residue of *Cp*OGA is colored *cyan*. The catalytic nucleophile is marked by a *circle. O*-GlcNAc, *magenta*; hTab1, *dark teal. C*, *Tt*OGA active site conservation. Surface residues that are identical in *Tt*OGA and hOGA are colored *dark blue*, and functionally conserved residues are colored *light blue. Numbers* represent the sites relative to the *O*-GlcNAc site. Presented in the *lower panel* is *Cp*OGA (Protein Data Bank code 2YDS). Surface residues that are unique to the α10-α11 loop of *Cp*OGA are colored *cyan*.

Previous work has shown that mutation of the *Cp*OGA catalytic acid Asp^298^ to asparagine (Asp^120^ in *Tt*OGA) results in loss of activity, but the enzyme retains the ability to bind to substrates (27). *Tt*OGA possessing an equivalent mutation (D120N) was utilized to trap a complex of *Tt*OGA with a glycopeptide derived from the validated hTab1 glycosylation site (VPY**gS**SAQ) (59). Synchrotron diffraction data were collected to 2.06 Å, and the structure was solved by molecular replacement (Table 1). Although the structure of hOGA is unknown, sequence alignments with structurally characterized bacterial homologues (*Cp*OGA and *Og*OGA) show that hOGA possesses an N-terminal triosephosphate isomerase (TIM) barrel catalytic domain; a middle “stalk” region, which comprises an α-helical domain (hereafter helical bundle domain) interrupted with a ∼200-amino acid-long, low complexity region; and a putative histone acetyltransferase domain (Fig. 1*B*) (28, 38). The *Tt*OGA structure reveals a classic (β/α)_8_ barrel (TIM barrel) at the N terminus and a C-terminal helical bundle domain (Fig. 10*A*) resembling those found in other bacterial OGA homologues. The *Tt*OGA catalytic domain is similar to that of *Cp*OGA and *Og*OGA (Fig. 10; r.m.s.d. = 1.3 Å for 228 C^α^ atoms and 1.4 Å for 233 C^α^ atoms, respectively). The sequence alignments show that the *Tt*OGA TIM barrel domain closely matches that of *Og*OGA and hOGA both in residue length and conservation except for minor differences in the residue length of the α4-β5 loop and the β5-α5 loop (Fig. 3). *O*-GlcNAcases, similar to lysosomal β-hexosaminidases (GH20 family members), utilize a double displacement retaining mechanism involving the participation of the 2-acetamido group of the substrate as the catalytic nucleophile (61). In *Tt*OGA, the active site residues involved in this mechanism are conserved: the catalytic acid (Asp^120^ in *Tt*OGA) protonates the glycosidic bond, a neighboring aspartic acid (Asp^119^ in *Tt*OGA) stabilizes the conformation of the 2-acetamido group, and the developing positive charge on the oxazolinium intermediate and an aspartic acid side chain (Asp^228^ in *Tt*OGA) hydrogen bonds O4 and O6, stabilizing the formation of the transition state. All these residues are identical in *Tt*OGA and positioned similarly (Fig. 10, *B* and *C*). Furthermore, the *N*-acetyl moiety of the substrate is fixed via a van der Waals/stacking interaction, and O3 and O4 hydroxyls are hydrogen-bonded with residues that are identical between *Tt*OGA, *Cp*OGA, *Og*OGA, and hOGA (Figs. 3 and 10*B*). All the interacting residues occupy the same space in the active site compared with that of *Cp*OGA (Fig. 10, *B* and *C*) (maximum atomic shift of 0.6 Å between interacting atoms). Therefore, the structural characterization of *Tt*OGA is in agreement with the observed enzymatic activity and inhibition (Fig. 5).

The *Tt*OGA C-terminal helical bundle domain, which has been proposed to play a role in recognition of the protein component of the substrates (28), comprises five helices (α11–α15) that are connected to the GH84 domain with two shorter helices (α9 and α10) (Fig 10). The helical bundle domain of *Tt*OGA is structurally similar to that of *Cp*OGA and *Og*OGA (r.m.s.d. = 2.2 Å for 148 C^α^ atoms and 2.5 Å for 121 C^α^ atoms, respectively; Fig. 8*B*). All the α-helices and loops of the *Tt*OGA helical bundle domain match those of *Cp*OGA with the exception of the α10-α11 loop: *Cp*OGA has a nine-amino acid insertion (Fig. 3). Crucially, this extended loop of *Cp*OGA reaches into the active site and may play role in positioning of the protein component of *O*-GlcNAc proteins near the active site (Fig. 10, *B* and *C*). Sequence alignments show that this loop extension is also absent in hOGA (Fig. 3). *Tt*OGA has a putative substrate binding groove conserved with that of hOGA (Fig. 10*C*). A previous study using *Cp*OGA has revealed that the peptide components of different model *O*-GlcNAc peptides adopt similar conformations due to the steric hindrance caused by the extended α10-α11 loop carrying a bulky tryptophan and the sequence-independent π-π stacking interaction of the surface Tyr^168^ with the −1 and −2 amides of the peptide (37) (Fig. 10*B*). However, in the absence of the α10-α11 loop, *Tt*OGA recognizes the peptide component of the hTab1 glycopeptide by a sequence-independent hydrogen bond between Gly^230^ and the backbone carbonyl oxygen at the −3 subsite of the glycopeptide (Fig. 10*C*). Given the relatively better conservation between *Tt*OGA and hOGA at the substrate binding groove, the *Tt*OGA·hTab1-*O*-GlcNAc complex may provide an alternative or more accurate model to study hOGA-substrate interactions.

## Discussion

Protein *O*-GlcNAcylation is an emerging essential regulator of cell signaling. There is increasing evidence for the involvement of *O*-GlcNAc in regulation of protein activity through the interplay with phosphorylation as recently shown for DNA-methylating ten-eleven translocation (TET) proteins (62) or phosphorylation-independent mechanisms as in the case of arginine methyltransferase 1 (CARM1) (63). However, despite a growing list of known and predicted *O*-GlcNAcylated proteins, we have a very limited understanding of the selectivity of the *O*-GlcNAc cycling enzymes for the appropriate target proteins. Additionally, the progress in our understanding of this process is significantly hampered by the essential nature of protein *O*-GlcNAcylation in most multicellular organisms (15–17, 19, 32). A possible solution to this problem is to identify a reductionist model system. This approach assumes identification of a simple, genetically tractable organism expressing active *O*-GlcNAc cycling enzymes and with visible *O*-GlcNAc-dependent and non-lethal phenotypes. In recent years, significant work has been undertaken toward identification of such an organism. Through these investigations, functional *O*-GlcNAc systems were identified in zebrafish (16), *D. melanogaster* (19, 57), *Caenorhabditis elegans* (31), and most recently *Trichoplax adhaerens* (64), a basal placozoan. In this report, we expand this list by identification of the first complete bacterial *O*-GlcNAc cycling system in the thermophile *T. terrenum*. By the analysis of OGT and OGA homologues expressed throughout the growth of *T. terrenum*, we validated the *in silico* prediction of the existence of these intracellular proteins in the proteome of *T. terrenum*. We also solved the structures of both proteins by x-ray crystallography, which provided further evidence for correct identification of *Tt*OGT and *Tt*OGA as members of the GT41 and GH84 families, respectively. Finally, we demonstrated that *Tt*OGA is active against an *O*-GlcNAcylated hTab1-derived peptide and that treatment of *T. terrenum* with a precursor of an OGT inhibitor causes growth inhibition.

Throughout the course of our investigation, we were not successful in detecting *O*-GlcNAc-modified proteins within the proteome of *T. terrenum* or *in vitro* activity of the recombinant form of *Tt*OGT. In addition to the possibility that such proteins do not exist, there are a number of possible explanations why we were unable to detect *O*-GlcNAc proteins in this organism. First, protein *O*-GlcNAcylation is known to be a substoichiometric modification (65). Thus, it is possible that the overall concentration of *O*-GlcNAc proteins in *T. terrenum* at any given time might be substantially lower than the levels found in systems studied to date and thus below the detection threshold of current technology. Second, the structure of *Tt*OGT suggests that a conformational change might be required for its activation, and this may not have been induced under the conditions tested in our experiments. Third, if the *T. terrenum O*-GlcNAc system has evolved along an evolutionary path parallel to that of animal *O*-GlcNAcylation, the immunoblotting assays, based on antibodies raised against eukaryotic *O*-GlcNAc proteins/peptides, might lack the specificity to detect *O*-GlcNAc proteins from the *T. terrenum* proteome. Finally, it is possible that this modification does not take place under the experimental conditions used in this study.

To further strengthen our hypothesis that *Tt*OGT plays an important role in the biology of *T. terrenum*, we took a physiological approach where the bacterial cells were treated with the cell-permeable precursor of the OGT inhibitor UDP-5S-GlcNAc. This treatment resulted in a complete inhibition of *T. terrenum* growth with a half-maximal effective concentration of 500 μm (Fig. 6). This is a concentration 2–50-fold higher than the precursor concentration used to inhibit protein *O*-GlcNAcylation in mammalian cells used in previous studies (66). However, this can be caused by differences in the efficiency of the biochemical pathways required for conversion of Ac_4_-5S-GlcNAc to the active form. Indeed, all components of the hexosamine biosynthetic pathway are annotated within the genome of *T. terrenum*; thus, we predict that UDP-5S-GlcNAc can be successfully synthesized from the precursor compound. Furthermore, we confirmed that Ac_4_-5S-GlcNAc is not toxic to the bacterial cells in general by subjecting another Gram-positive bacterium, *B. subtilis*, to the same doses of the compound with no visible impact on the growth kinetics (Fig. 6*C*). By means of electron microscopy, we confirmed that Ac_4_-5S-GlcNAc does not affect the synthesis of peptidoglycan (Fig. 7), which would be another likely manifestation of an off-target activity of UDP-5S-GlcNAc in *T. terrenum*.

The second of the *O*-GlcNAc cycling enzyme homologues, *Tt*OGA, is active in a range of OGA-specific assays. Like many other bacterial OGA homologues, *Tt*OGA was initially annotated as a hyaluronidase. However, we did not detect any activity of *Tt*OGA against hyaluronic acid in the conditions in which we could observe a clear *O*-GlcNAcase activity. Furthermore, *Tt*OGA is inhibited by the OGA-specific inhibitor GlcNAcstatin G (Fig. 5*B*) with a *K_i_* similar to that for the human and *C. perfringens* OGAs. We concluded that the enzymatic parameters of *Tt*OGA are comparable with those of characterized OGAs. The structural data further confirm the high level of structural conservation between *Tt*OGA and *Cp*OGA, and sequence alignments suggest that, in terms of loop structure and the peptide binding groove, *Tt*OGA is a better structural model for hOGA.

From the data presented in this report, we concluded that *T. terrenum* is the first bacterium that fulfils the theoretical requirements of possessing a functional protein *O*-GlcNAcylation system. The organism encodes an active *O*-GlcNAcase and an *O*-GlcNAc-transferase that, from the structure, appear to be catalytically competent. We predict that the low abundance of *O*-GlcNAcylated proteins and a different evolutionary origin of the *T. terrenum* system are the reasons for the apparent absence of *O*-GlcNAcylated proteins in *T. terrenum*. The phylogenetic isolation of the *T. terrenum O*-GlcNAcylation system presents an interesting question of the selection pressure that may have driven the acquisition of this system as no proteins similar on the peptide sequence level to *Tt*OGT or *Tt*OGA can be found in the published genomes of the most closely related taxon, Chloroflexi (67). *Tt*OGT and *Tt*OGA may have been acquired by two individual horizontal gene transfer events as these enzymes are encoded on separate chromosomes.

Interestingly, protein *O*-GlcNAcylation was demonstrated not only to take part in the regulatory circuitry by interaction with protein phosphorylation (20), but some reports also postulate that *O*-GlcNAcylation contributes to increased protein stability (68) and that this effect might be a response to increased temperatures (69). It is possible to speculate that, in the case of *T. terrenum*, acquisition of the protein *O*-GlcNAcylation system was driven by the requirement to adapt to its natural environment of temperatures between 50 and 90 °C (41) and protect its proteome from these high temperatures. Despite the fact that genetic modification of *T. terrenum* is not yet possible, we believe that further study of this post-translational protein modification new to the Bacteria kingdom will allow for better understanding of the *O*-GlcNAc system in multicellular organisms and might eventually lead to the development of a reductionist system to study protein *O*-GlcNAcylation based on *T. terrenum*.

## Author Contributions

D. M. F. v. A. conceived the project. A. O., M. G., and D. M. F. v. A. designed the experiments and analyzed the data. A. O. performed cell biology experiments. M. G. performed enzymology and crystallography experiments. A. T. F. performed all cloning. A. A. L., A. O., M. G., and D. M. F. v. A. wrote the paper. All authors reviewed the results and approved the final version of the manuscript.

## Footnotes

4 The abbreviations used are:

*O*-GlcNAc: *O*-linked *N*-acetylglucosamine
OGT: *O*-GlcNAc-transferase
OGA: *O*-GlcNAcase
TPR: tetratricopeptide repeat
*Cp*: *C. perfringens*
*Tt*: *T. terrenum*
DMSO: dimethyl sulfoxide
4MU-GlcNAc: 4-methylumbelliferyl-*N*-acetyl-β-d-glucosaminide
hTab1: human TAK1-binding protein 1
GT41: glycosyltransferase 41
GH84: glycoside hydrolase 84
hOGT: human OGT
hOGA: human OGA
Ac_4_-5S-GlcNAc: peracetylated 5S-GlcNAc
r.m.s.d.: root mean square deviation
TLR: tetratricopeptide-like region
*Og*: *O. granulosus*.
